# Impact of Operative Timing in Infective Endocarditis with Cerebral Embolism—The Risk of Intermediate Deterioration

**DOI:** 10.3390/jcm10102136

**Published:** 2021-05-15

**Authors:** Alexey Dashkevich, Georg Bratkov, Yupeng Li, Dominik Joskowiak, Sven Peterss, Gerd Juchem, Christian Hagl, Maximilian Luehr

**Affiliations:** 1 Department of Cardiac Surgery, University Hospital, LMU Munich, 81377 Munich, Germany; alexey.dashkevich@med.uni-muenchen.de (A.D.); bratkov@gmx.de (G.B.); Dominik.Joskowiak@med.uni-muenchen.de (D.J.); Sven.Peterss@med.uni-muenchen.de (S.P.); Gerd.juchem@med.uni-muenchen.de (G.J.); Christian.Hagl@med.uni-muenchen.de (C.H.); 2 Department of Political Science and Economics, Rowan University, Glassboro, NJ 08028, USA; liy@rowan.edu; 3 Department of Cardiothoracic Surgery, Heart Center of the University of Cologne, 50937 Cologne, Germany

**Keywords:** infective endocarditis (IE), cerebral embolism, ischemic stroke, hemorrhagic stroke, mitral valve, aortic valve, surgical timing

## Abstract

Cerebral embolism due to infective endocarditis (IE) is associated with significant morbidity and mortality. The optimal time-interval between symptomatic stroke and cardiac surgery remains unclear. This study aimed to analyze the patients’ outcomes and define the potential risk factors with regard to surgical timing for IE patients with preoperative symptomatic cerebral embolism (CE). A total of 119 IE patients with CE were identified and analyzed with regard to operative timing: early (1–7 days), intermediate (8–21 days), and late (>22 days). The preoperative patient data, comorbidities and previous cardiac surgical procedures were analyzed to identify potential predictors and independent risk factors for in-hospital mortality using univariate and multivariate regression analysis. Actuarial survival was estimated by the Kaplan-Meier method. In-hospital mortality for the entire study cohort was 15.1% (*n* = 18), and in comparison, between groups was found to be highest in the intermediate surgical group (25.7%). Univariate analysis identified preoperative mechanical ventilation dependent respiratory insufficiency (*p* = 0.006), preoperative renal insufficiency (*p* = 0.019), age (*p* = 0.002), large vegetations (*p* = 0.018) as well as intermediate (*p* = 0.026), and late (*p* = 0.041) surgery as predictors of in-hospital mortality. The presence of large vegetations (>8 mm) (*p* = 0.019) and increased age (*p* = 0.037)—but not operative timing—were identified as independent risk factors for in-hospital mortality. In the presence of large vegetations (>8 mm), cardiac surgery should be performed early and independently from the entity of cerebral embolic stroke. Postponing surgery to achieve clinical stabilization and better postoperative outcomes of IE patients with CE is reasonable, however, worsening of the disease process with deterioration and resulting heart failure during the first 3 weeks after CE results in a significantly higher in-hospital mortality and inferior long-term survival.

## 1. Introduction

Infective endocarditis (IE) can lead to central or peripheral embolic events in up to 50% of all IE patients [1,2,3]. Cerebral embolism with resulting stroke may occur in 15–30% of patients and, therefore, represents one of the most severe complications of IE associated with significantly increased morbidity and mortality [1,4]. However, the clinical manifestations in IE patients may vary widely and depend on the localization, extent, and underlying etiology of cerebral embolism (hemorrhagic, ischemic or non-ischemic). Surgery for IE in patients with neurological events should be performed without delay if indicated [5], e.g., presence of silent embolism or transient ischemic attack (class of recommendation I, level of evidence B according to the guidelines of the European Society of Cardiology, ESC) [4]. In the presence of severe preoperative neurological damage or intracranial hemorrhage, the operation should be postponed [6,7]. However, the optimal time interval between symptomatic cerebral embolism due to IE and cardiac surgery remains unclear (class of recommendation IIa, level of evidence B [4]). Delay of cardiac surgery in the presence of preoperative cerebral hemorrhage for at least 4 weeks is a living practice, as the risk of repetitive cerebral embolism has been reported to decrease over time with broad-spectrum antibiotic treatment [5]. Even delay of surgery for one week was described to be favorable for the patient’s outcome in these cases [8]. On the other side, several studies indicate acceptable patient outcomes for early surgery, by means of only a few days after the occurrence of cerebral hemorrhage due to embolism [9,10,11,12]. However, clinical decision-making often remains arbitrary and needs careful personalized patient assessment, especially in the case of hemorrhagic stroke due to embolism [13]. It was the purpose of this study to analyze the perioperative patient outcomes and to define potential risk factors with regard to the timing of cardiac surgery for IE patients with symptomatic cerebral embolism.

## 2. Materials and Methods

The perioperative data of 119 consecutive patients (78 males; 65.6%) suffering from IE with preoperative cerebral embolism were retrospectively analyzed between January 2005 and December 2018. Three study groups were defined for statistical analysis with regard to operative timing after cerebral embolism: early (1–7 days), intermediate (8–21 days), and late (>22 days). Table 1 shows the preoperative patient characteristics at the time of admission to our institution.

### Definitions and Statistical Analysis

The Ethics Committees of the Ludwig Maximilian University Munich granted approval for the study (#19-730). The data acquisition was performed by retrospective chart review. Further information was gathered at the time of routine follow-up at our clinical center or via a telephone interview with the patients and/or their respective physicians. The overall follow-up time comprised the time interval between the primary surgery for IE and the latest contact with the respective patient. No expired patient was excluded. In-hospital mortality was defined as death before hospital discharge. Follow-up mortality was defined as death after hospital discharge. Postoperative renal failure was defined either as temporary or permanent with regard to the need for dialysis. The definition of preoperative cerebral embolism, either as hemorrhagic stroke, ischemic stroke, or non-hemorrhagic/non-ischaemic embolism, was performed by a single neurosurgeon blinded to the study. New postoperative stroke was defined as any new cerebral infarction diagnosed and verified by neurologic assessment and/or computed tomography of the brain. The data analysis was performed according to the statistical and data reporting guidelines of the European Journal of Cardio-Thoracic Surgery [14]. Categorical variables were reported using the number and percentage of occurrences. Continuous variables were expressed as mean ± standard deviation (SD) or (if SD exceeded the mean value) as median with interquartile range (IQR; 25th to 75th percentile). The Fisher–Freeman–Halton test was used for group comparison (Table 1, Table 2, Table 3 and Table 4). The preoperative data on patient characteristics, comorbidities, and previous cardiac surgical procedures were analyzed to identify potential predictors and independent risk factors for in-hospital mortality using univariate and multivariate regression analysis. A binary logistic regression model was built using the variables with a *p*-value < 0.1 from univariate analysis (Table 5). A *p*-value of < 0.05 was considered statistically significant. Actuarial survival was estimated by the Kaplan–Meier method with the Log-rank (Mantle-Cox) and the Gehan–Breslow–Wilcoxon tests for group comparison. The statistical analysis was performed with the SPSS statistical software package (version 25.0; IBM, Ehningen, Germany).

## 3. Results

Between January 2005 and December 2018, 119 patients suffering from native IE (*n* = 89) and prosthetic valve endocarditis (PVE; *n* = 30) of the aortic, mitral, or tricuspid valve with preoperative cerebral embolism were operated on at our institution. Infection involved the aortic valve in 68 (57.1%), the mitral valve in 67 (56.3%), and/or the tricuspid valve in 2 (1.7%) patients. Computed tomography showed preoperative cerebral embolism in all patients, including ischemic stroke in 79 (66.4%), hemorrhagic stroke in 29 (24.4%), or non-hemorrhagic/non-ischemic embolism in 11 (9.2%) patients. Acute neurological symptoms were preoperatively evident in 85 (71.4%) patients. In comparison to the early surgery group, the incidence of hemorrhagic stroke (38.6% vs. 10.0%, *p* = 0.009) was significantly higher in the intermediate and late surgical groups. The incidence aortic valve endocarditis was highest in the early surgery group (*n* = 28; 70%; *p* = 0.019), while patients in the intermediate group had the most cases of PVE (*n* = 17; 48.6%; *p* = 0.003) and cardiac reoperations (*n* = 16; 45.7%; *p* = 0.003) (Table 1).

Preoperatively obtained blood cultures revealed positive results in 93 patients (78.2%) with Staphylococcus spp. (42.0%), Streptococcus spp. (18.5%) and Enterococcus spp. (13.5%) as the most frequently identified pathological strains without statistical difference in distribution between groups. Other pathogens we only identified in 5 patients of the late surgical group (11.4%; *p* = 0.011) (Table 1).

The indication for surgical treatment was made due to progression of a single or a combination of preoperative complications such as valve dysfunction (regurgitation) (*n* = 102; 85.7%), paravalvular abscess formation (*n* = 31; 26.0%), cardiac low output syndrome in (*n* = 6; 5.0%) or large valve vegetations (>8 mm) (*n* = 57; 47.9%). In comparison, aortic regurgitation was significantly less present in the late surgical group (*p* = 0.014). Development of preoperative low cardiac output syndrome was significantly higher in the intermediate group (*p* = 0.007) (Table 2).

Surgical treatment of infective endocarditis was performed in an elective (47.9%), urgent (24.4%), or emergent (27.7%) clinical setting. The aortic valve was directly replaced in all cases using 45 biological and 4 mechanical valves (*n* = 49; 41.2%), while 3 patients received aortic valve replacement by homograft (*n* = 3; 2.5%). The mitral valve was directly replaced in 41 (34.5%) patients using 37 biological and 4 mechanical valves, while 8 (6.7%) patients received a mitral repair. The tricuspid valve was directly replaced in 2 (1.7%) patients using 1 biological and 1 mechanical valve, respectively. Two (1.7%) patients received a tricuspid repair. Other concomitant procedures comprised left ventricular outflow tract reconstruction by pericardial patch plasty (*n* = 22; 18.5%), aortic surgery (*n* = 6; 5%), coronary artery bypass grafting (*n* = 12; 10.1%), ventricular septal defect (VSD) closure (*n* = 3; 2.5%), pacemaker explantation (*n* = 4; 3.4%), and other minor procedures (*n* = 6; 5.0%). Group comparison revealed that patients in the early group were operated significantly more often in an emergent setting than in the intermediate or late surgical groups (*p* = 0.001). Moreover, reconstructive surgery of the left ventricular outflow tract was also more often required in the early group than in the other two groups. On the other hand, patients operated on late significantly more often required mitral valve replacement than those in the intermediate group (*p* = 0.022). No differences were found regarding the operatives times between the three groups. The details of intraoperative data are given in Table 3.

The in-hospital and follow-up mortality rates for the entire study cohort were 15.1% (*n* = 18) and 10.1% (*n* = 12). No significant difference with regard to in-hospital mortality was found by comparison the three groups (*p* = 0.066). However, in-hospital mortality was highest in the intermediate (*n* = 9; 25.7%) and early surgical group (*n* = 6; 15.0%). During follow-up, mortality rates between the three groups (early vs. intermediate vs. late) were comparable (*p* = 0.801) (Table 4).

**Figure 1:**
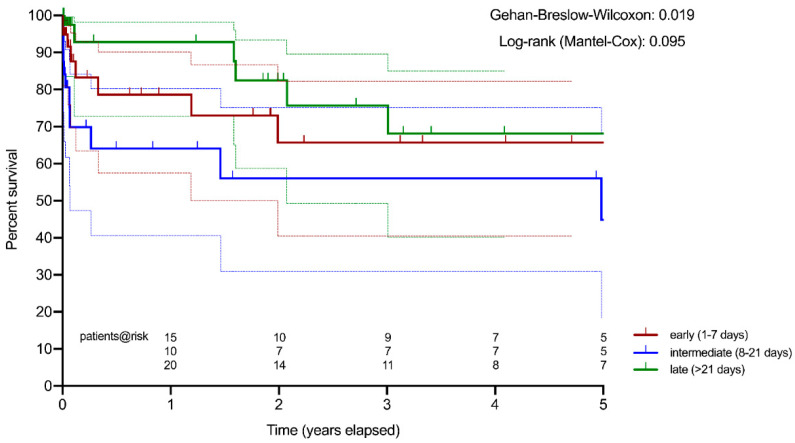
Actuarial survival estimation (Kaplan–Meier): early surgery (red) vs. intermediate surgery (blue) vs. late surgery (green).

Actuarial survival estimation of patients after intermediate surgery was significantly worse compared to the early and late surgical groups (Gehan–Breslow–Wilcoxon test: *p* = 0.019) (Figure 1).

The most frequent postoperative complications were noted as permanent renal failure (*n* = 26; 21.9%), sepsis (*n* = 28; 23.5%), hemorrhage with need for re-thoracotomy (*n* = 11; 9.2%), need for secondary pacemaker implantation (*n* = 11; 9.2%), and low cardiac output syndrome (*n* = 10; 8.4%). New postoperative stroke occurred in 7 patients (5.9%) after hemorrhagic (*n* = 2) or ischemic stroke (*n* = 5). Sternal wound infection only occurred in 2.5% (*n* = 3) of all patients. The incidence of postoperative 9 sepsis/SIRS was significantly higher in the intermediate group (*n* = 13) if compared to the early and late surgical groups (*p* = 0.049). No significant differences between the three groups were found with regard to median ventilation time (18 h), intensive care unit (5 days), and hospital stay (19 days). The data on postoperative outcomes are summarized in Table 4.

Univariate analysis using the preoperative patient characteristics, comorbidities and previous cardiac surgical procedures of the entire study cohort (*n* = 119) identified “age” (*p* = 0.002), “presence of large vegetations (>8 mm)” (*p* = 0.018), “preoperative renal insufficiency” (*p* = 0.019), “previous mechanical ventilation” (*p* = 0.006), as well as intermediate (*p* = 0.026) and late surgery (*p* = 0.041) as predictors of in-hospital mortality. However, only the preoperative parameters “presence of large vegetations (>8 mm)” (OR 9.408; 95%-CI: 1.455–60.821; *p* = 0.019) and “increased age” (OR 1.100; 95%-CI: 1.006-1.202; *p* = 0.037) were identified as independent risk factors for in-hospital mortality. The data on uni and multivariate analyses to identify risk factors for in-hospital mortality are summarized in Table 5.

## 4. Discussion

Cerebral embolism represents a severe complication of infective endocarditis (IE) but does not alter the indication for surgical treatment [1]. As no clear recommendation for the optimal time interval between the initial occurrence of cerebral embolism due to IE and surgical treatment exists, the aim of this study was to analyze the patients’ outcomes with regard to the timing of cardiac surgery. For this purpose, we divided the patients into three study groups depending on the surgical time point (early, intermediate, and late).

In our study, the in-hospital mortality rate of all patients operated for IE after cerebral embolism was 15.1% and is well comparable to the results reported in the literature [4]. Interestingly, early surgery did not result in an inferior outcome despite a significantly higher number of urgent and emergent cases. However, open surgery in the intermediate time interval (8–21 days) had the worst postoperative outcome, especially if compared to patients operated after 3 weeks (25.7 vs. 6.8%). Despite the fact that the follow-up mortality rates were not significantly different between the three groups, survival estimation by the Kaplan–Meier method clearly showed a significantly superior long-term survival for patients operated after 3 weeks (late) compared to the cases operated in the intermediate interval (8–21 days).

In case of silent stroke or transient ischaemic attack (TIA) a delay in open surgery is not always necessary [10]. However, after symptomatic stroke due to cerebral embolism due to IE, the decision towards surgery is dependent on the severity of neurological damage and the general neurological prognosis [15]. The optimal timing for cardiac surgery after an embolic stroke still remains unclear, as different timing strategies have been controversially discussed in the current literature. As reported, early surgery for patients with either ischaemic [10,15] or hemorrhagic [12] cerebral embolism showed comparable results to postponed surgical patients with hemorrhagic embolism for at least 1 month [16,17].

In our study, early operation—within the first week after cerebral embolism—was not generally inferior in terms of in-hospital mortality if compared to the intermediate and late surgical groups. This might be explained by the fact that the incidence of previous cardiac surgery and prosthetic valve endocarditis were significantly lower in this group. Kim et al. reported early surgery (if indicated) to be safe for patients with IE and neurological complications if the cerebral embolus has a diameter of under 20 mm [18]. Moreover, patients in the intermediate and late surgical groups showed higher incidences of hemorrhagic stroke and acute preoperative neurological symptoms. Thus, patients with evident neurological symptoms and intracranial hemorrhage were predominantly postponed for surgery in our clinical setting, which is common clinical practice [16,17]. Further, we found intermediate surgery to result in higher in-hospital mortality and significantly inferior long-term survival compared to open cardiac surgery postponed for at least 3 weeks. The inferior outcome of those patients seems to be related to an acute progression of the disease process of IE and worsening of heart failure, as the incidence of relevant aortic regurgitation (65.7% vs. 36.4%), preoperative low cardiac output syndrome (14.3% vs. 0%), peripheral septic embolism (54.3% vs. 38.6%), and postoperative sepsis (37.1% vs 15.9%) and permanent renal failure (31.4% vs. 11.4%) were found to be higher in the intermediate than in the late surgical group.

In the univariate analysis, several predictors of in-hospital mortality were identified for the entire study group. Such predictors as preoperative mechanical ventilation dependent respiratory insufficiency, dialysis-dependent renal insufficiency, and age can be recognized as severe comorbidities or factors worsening a patient’s preoperative status and per se increasing the overall risk of cardiac surgery. As demonstrated recently, a valve vegetation size of >20 mm has been clearly associated with a higher mortality rate due to the imminent risk of cerebral embolization [19]. Multivariate regression analysis of our data identified the presence of large vegetations (>8 mm) and increased age—but not the operative timing—as independent risk factors for in-hospital mortality. Further, the rate of new permanent postoperative stroke was moderate with 5.9% and was not different in patients with hemorrhagic or ischaemic preoperative cerebral embolism. Thus, the authors suggest that in the presence of large vegetations of more than 8 mm, open cardiac surgery should be performed early and independently from the entity and severity of cerebral embolic stroke.

Clear recommendations regarding the surgery timing after cerebral embolism are currently missing in the 2015 ESC guidelines due to a lack of scientific evidence [4]. As the large prospective clinical trials are barely possible in this comprehensive patient collective, national or international endocarditis registries are required to better understand the clinical patterns, to improve the time flow of surgical treatment, and to optimize the neurological outcome of the patients suffering from cerebral embolism.

## 5. Conclusions

The outcomes of early surgery after cerebral embolism in patients with IE are acceptable in experienced centers. In the presence of large vegetations (>8 mm), open cardiac surgery should be performed early and independently from the entity of cerebral embolic stroke. Postponing surgery to achieve clinical stabilization and better postoperative outcomes of IE patients with cerebral embolism is reasonable, as long as the patient remains stable throughout the intermediate phase. However, worsening of the disease process and resulting heart failure during the first 3 weeks after cerebral embolism results in a higher in-hospital mortality and significantly inferior long-term survival.

## Figures and Tables

**Table 1:** Patient characteristics (*n* = 119).

Parameters, *n* (%)	All Patients (*n* = 119)	Early (1–7 Days) (*n* = 40)	Intermediate (8–21 Days) (*n* = 35)	Late (>22 Days) (*n* = 44)	*p*-Value
Age (mean, years ± SD)	61.0 ± 14.7	57.5 ± 17.3	62.5 ± 14.3	62.9 ± 12.1	0.358
Male gender	78 (65.5%)	24 (60.0%)	26 (74.3%)	28 (63.6%)	0.407
AV endocarditis	68 (57.1%)	28 (70.0%)	22 (62.9%)	18 (40.9%)	0.019 *
MV endocarditis	67 (56.3%)	20 (50.0%)	18 (51.4%)	29 (65.9%)	0.268
TV endocarditis	2 (1.7%)	1 (2.5%)	1 (2.9%)	-	0.530
Prev. cardiac surgery	33 (27.7%)	6 (15.0%)	17 (48.6%)	10 (22.7%)	0.003 *
PVE	30 (25.2%)	5 (12.5%)	16 (45.7%)	9 (20.5%)	0.003 *
Smoking history	23 (19.3%)	9 (22.5%)	3 (8.6%)	11 (25.0%)	0.152
Coronary artery disease	29 (24.4%)	7 (17.5%)	9 (25.7%)	13 (29.6%)	0.399
Arterial hypertension	73 (61.3%)	21 (52.5%)	24 (68.6%)	28 (63.6%)	0.335
Pulmonal hypertension	8 (6.7%)	2 (5.0%)	2 (5.7%)	2 (4.6%)	0.815
Diabetes mellitus	25 (21.0%)	6 (15.0%)	9 (25.7%)	10 (22.7%)	0.493
Hyperlipoproteinemia	36 (30.3%)	11 (27.5%)	12 (34.3%)	13 (29.6%)	0.809
Obesity	23 (19.3%)	4 (10.0%)	8 (22.9%)	11 (25.0%)	0.181
Peripheral artery disease	8 (6.7%)	2 (5.0%)	5 (14.3%)	1 (2.3%)	0.132
Splenomegaly	13 (10.9%)	3 (7.5%)	4 (11.4%)	6 (13.6%)	0.708
LVEF < 35%	10 (8.4%)	3 (7.5%)	4 (11.4%)	3 (6.8%)	0.772
Renal insufficiency	42 (35.3%)	16 (40.0%)	13 (37.1%)	13 (29.6%)	0.584
Dialysis dependent	6 (5.0%)	2 (5.0%)	3 (8.6%)	1 (2.3%)	0.438
COPD	13 (10.9%)	4 (10.0%)	3 (8.6%)	6 (13.6%)	0.814
Cerebral embolism					
Ischemic stroke	79 (66.4%)	31 (77.5%)	22 (62.9%)	26 (59.1%)	0.177
Haemorrhagic stroke	29 (24.4%)	4 (10.0%)	8 (22.9%)	17 (38.6%)	0.009 *
Non-isch./-haemorrhagic	11 (9.2%)	5 (12.5%)	5 (14.3%)	1 (2.3%)	0.103
Prev. mechanical vent.	20 (16.8%)	7 (17.5%)	7 (20.0%)	6 (13.6%)	0.746
Preop. acute neurology	85 (71.4%)	23 (57.5%)	27 (77.1%)	35 (79.6%)	0.055
Causative pathogens					
Staphylococcus spp.	50 (42.0%)	19 (47.5%)	15 (42.9%)	16 (36.4%)	0.582
Streptococccus spp.	22 (18.5%)	8 (20.0%)	5 (14.3%)	9 (20.5%)	0.747
Enterococcus spp.	16 (13.5%)	4 (10.0%)	7 (20.0%)	5 (11.4%)	0.439
Other	5 (4.2%)	-	-	5 (11.4%)	0.011 *

CABG = coronary artery bypass graft; PM = pacemaker; HLM = heart lung machine; IQR = interquartile range; ICU = intensive care unit; COPD = chronic obstructive pulmonary disease; LVEF = left ventricular ejection fraction; AV = aortic valve; MV = mitral valve; TV = tricuspid valve; SD = standard deviation (* = statistically significant).

**Table 2:** Indications for surgery (*n* = 119).

Parameters, *n* (%)	All Patients (*n* = 119)	Early (Group 1) (*n* = 40)	Intermediate (Group 2) (*n* = 35)	Late (Group 3) (*n* = 44)	*p*-Value
Aortic regurgitation	64 (53.8%)	25 (62.5%)	23 (65.7%)	16 (36.4%)	0.014 *
I	22 (18.5%)	5 (12.5%)	10 (28.6%)	7 (15.9%)	0.173
II	16 (13.5%)	8 (20.0%)	6 (17.1%)	2 (4.6%)	0.074
III	24 (20.2%)	12 (30.0%)	6 (17.1%)	6 (13.6%)	0.165
IV	1 (0.8%)	-	1 (2.9%)		0.297
Mitral regurgitation	89 (74.8%)	32 (80.0%)	22 (62.9%)	35(79.6%)	0.154
I	36 (30.3%)	13 (32.5%)	11 (31.4%)	12 (27.3%)	0.859
II	27 (22.7%)	11 (27.5%)	6 (17.1%)	10 (22.7%)	0.565
III	16 (13.5%)	5 (12.5%)	3 (8.6%)	8 (18.2%)	0.465
IV	8 (6.7%)	3 (7.5%)	1 (2.9%)	4 (9.1%)	0.590
Tricuspid regurgitation	32 (26.9%)	10 (25.0%)	11 (31.4%)	11 (25.0%)	0.771
I	25 (1.7%)	8 (20.0%)	9 (25.7%)	8 (18.2%)	0.703
II	3 (2.5%)	-	-	3 (6.8%)	0.108
III	4 (3.4%)	2 (5.0%)	2 (5.7%)	-	0.320
Low cardiac output	6 (5.0%)	1 (2.5%)	5 (14.3%)	-	0.007 *
Abscess formation (echo)	31 (26.0%)	11 (27.5%)	10 (28.6%)	10 (22.7%)	0.814
Valve vegetations (echo)					
<5 mm	22 (18.5%)	5(12.5%)	8(22.9%)	9(20.5%)	0.432
5–8 mm	17 (14.3%)	9(22.5%)	4(11.4%)	4(9.1%)	0.216
>8 mm	57 (47.9%)	19(47.5%)	17(48.6%)	21(47.7%)	0.956
Peripheral septic embolism	55 (46.2%)	19 (47.5%)	19 (54.3%)	17 (38.6%)	0.183

(* = statistically significant).

**Table 3:** Intraoperative data (*n* = 119).

Parameters, *n* (%)	All Patients (*n* = 119)	Early (Group 1) (*n* = 40)	Intermediate (Group 2) (*n* = 35)	Late (Group 3) (*n* = 44)	*p*-Value
**Priority**					
Elective	57 (47.9%)	11 (27.5%)	21 (60.0%)	25 (56.8%)	0.006 *
Urgent	29 (24.4%)	9 (22.5%)	8 (22.9%)	12 (27.3%)	0.852
Emergent	33 (27.7%)	20 (50.0%)	6 (17.1%)	7 (15.9%)	0.001 *
**Surgical procedures**					
Aortic valve					
Replacement	49 (41.2%)	21 (52.5%)	12 (34.3%)	16 (36.4%)	0.199
Homograft	3 (2.5%)	1 (2.5%)	2 (5.7%)	-	0.195
Mitral valve					
Replacement	41 (34.5%)	11 (27.5%)	5 (14.3%)	21 (47.7%)	0.022 *
Repair	8 (6.7%)	3 (7.5%)	2 (5.7%)	3 (6.8%)	1.000
Tricuspid valve					
Replacement	2 (1.7%)	1 (2.5%)	1 (2.7%)	-	0.530
Repair	2 (1.7%)	-	2 (5.7%)	-	0.085
Aortic surgery					
Asc. replacement	5 (4.2%)	-	1 (2.7%)	4 (9.1%)	0.261
Hemiarch	1 (0.8%)	-	-	1 (2.3%)	1.000
CABG	12 (10.1%)	2 (5.0%)	5 (14.3%)	5 (11.4%)	0.375
Pericardial patch plasty	22 (18.5%)	11 (27.5%)	3 (8.6%)	8 (18.2%)	0.108
VSD closure	3 (2.5%)	-	1 (2.7%)	2 (4.6%)	0.512
PM explantation	4 (3.4%)	1 (2.5%)	1 (2.7%)	2 (4.6%)	1.000
Other procedures	6 (5.0%)	-	1 (2.7%)	5 (11.4%)	0.101
**Intraoperative data, mean (±SD)**					
HLM time (min)	138.2 ± 61.1	137.0 ± 63.8	146.6 ± 58.6	132.5 ± 61.1	0.398
Cross-clamp time (min)	94.8 ± 41.9	93.5 ± 40.6	98.7 ± 38.0	92.9 ± 46.6	0.584
Reperfusion time (min)	36.0 ± 20.6	35.8 ± 22.7	37.6 ± 20.1	35.0 ± 19.2	0.833
Lowest temperature (°C)	33.0 ± 3.5	33.0 ± 3.6	32.0 ± 3.3	33.0 ± 3.5	0.670

CABG = coronary artery bypass graft; VSD = ventricular septal defect; PM = pacemaker; HLM = heart lung machine; IQR = interquartile range; ICU = intensive care unit; SD = standard deviation (* = statistically significant).

**Table 4:** Postoperative outcome (n = 119).

Parameters, *n* (%)	All Patients (*n* = 119)	Early (Group 1) (*n* = 40)	Intermediate (Group 2) (*n* = 35)	Late (Group 3) (*n* = 44)	*p*-Value
**Mortality, *n* (%)**					
In-hospital	18 (15.1%)	6 (15.0%)	9 (25.7%)	3 (6.8%)	0.066
During Follow-up	12 (10.1%)	3 (7.5%)	4 (11.4%)	5 (11.4%)	0.801
**Postoperative complications, *n* (%)**					
Reintubation	11 (9.2%)	4 (10.0%)	5 (14.3%)	2 (4.6%)	0.322
Tracheostomy	6 (5.0%)	3 (7.5%)	2 (5.7%)	1 (2.3%)	0.581
Renal failure (temp.)	7 (5.9%)	3 (7.5%)	3 (8.6%)	1 (2.3%)	0.384
Renal failure (perm.)	26 (21.9%)	10 (25.0%)	11 (31.4%)	5 (11.4%)	0.080
New stroke (perm.)	7 (5.9%)	4 (10.0%)	2 (5.7%)	1 (2.3%)	0.331
LCO syndrome	10 (8.4%)	3 (7.5%)	5 (14.3%)	2 (4.6%)	0.288
Sepsis/SIRS	28 (23.5%)	8 (20.0%)	13 (37.1%)	7 (15.9%)	0.049 *
Postop. PM	11 (9.2%)	2(5.0%)	4 (11.4%)	5 (11.4%)	0.523
Rethoracotomy	11 (9.2%)	3 (7.5%)	4 (11.4%)	4 (9.1%)	0.861
Inferior pericardiotomy	9 (7.6%)	4 (10.0%)	1 (2.9%)	4 (9.1%)	0.466
Sternal wound infection	3 (2.5%)	1 (2.5%)	2 (5.7%)	-	0.193
**Hospital and ICU stay, mean (±SD)**					
Ventilation (hours; IQR)	18.0 (9.8 53.3)	24.0 (12.0–72.0)	20.1 (9.3–75.0)	14.0 (9.0–26.0)	0.209
ICU stay (days)	5.5 ± 6.1	6.2 ± 6.0	6.2 ± 6.8	4.3 ± 5.4	0.213
Hospital stay (days)	18.8 ± 14.1	15.6 ± 10.5	17.1 ± 11.1	23.2 ± 17.8	0.210

ECMO/ECLS = extracorporeal membrane oxygenation/extracorporeal life support; LCO = low cardiac output; SIRS = systemic inflammatory response syndrome; PM = pacemaker; IQR = interquartile range; ICU = intensive care unit. (* = statistically significant).

**Table 5:** Uni- and multivariate analysis to identify risk factors for in-hospital mortality.

Univariate Analysis				*p*-Value
Increased age				0.002 *
Coronary artery disease				0.097
Previous CABG				0.059
Vegetations >8 mm				0.018 *
NYHA IV				0.065
Low cardiac output				0.060
Preoperative renal insufficiency				0.019 *
Dialysis (preoperative)				0.059
Previous mechanical ventilation				0.006 *
Time-to-operation (after cerebral embolization)				
Intermediate surgery (8–21 days)				0.026 *
Late surgery (≥22 days)				0.041 *
95%-Confidence interval				
**Multivariate analysis**	Odds ratio	low	high	*p*-value
Vegetations >8 mm	9.408	1.455	60.821	0.019 *
Increased age	1.100	1.006	1.202	0.037 *

CABG = coronary artery bypass graft; NYHA = New York Heart Association. (* = statistically significant).

## References

[1] Habib G., Erba P.A., Iung B., Donal E., Cosyns B., Laroche C., Popescu B.A., Prendergast B., Tornos P., Sadeghpour A. (2019). Clinical presentation, aetiology and outcome of infective endocarditis. Results of the ESC-EORP EURO-ENDO (European infective endocarditis) registry: A prospective cohort study. Eur. Heart J..

[2] Salaun E., Touil A., Hubert S., Casalta J.-P., Gouriet F., Robinet-Borgomano E., Doche E., Laksiri N., Rey C., Lavoute C. (2018). Intracranial haemorrhage in infective endocarditis. Arch. Cardiovasc. Dis..

[3] Hubert S., Thuny F., Resseguier N., Giorgi R., Tribouilloy C., Le Dolley Y., Casalta J.-P., Riberi A., Chevalier F., Rusinaru D. (2013). Prediction of Symptomatic Embolism in Infective Endocarditis. J. Am. Coll. Cardiol..

[4] Habib G., Lancellotti P., Antunes M.J., Bongiorni M.G., Casalta J.-P., Del Zotti F., Dulgheru R., El Khoury G., Erba P.A., Iung B. (2015). 2015 ESC Guidelines for the manage-ment of infective endocarditis: The Task Force for the Management of Infective Endocarditis of the European Society of Cardiology (ESC)Endorsed by: European Association for Cardio-Thoracic Surgery (EACTS), the European Association of Nuclear 284 Medicine (EANM). Eur. Heart J..

[5] García-Cabrera E., Fernández-Hidalgo N., Almirante B., Ivanova-Georgieva R., Noureddine M., Plata A., Lomas J.M., Galvez-Acebal J., Hidalgo-Tenorio C., Ruiz-Morales J. (2013). Neurologi-cal Complications of Infective Endocarditis: Risk Factors, Outcome, and Impact of Cardiac Surgery: A Multicenter Ob-servational Study. Circulation.

[6] Baddour L.M., Wilson W.R., Bayer A.S., Fowler V.G., Tleyjeh I.M., Rybak M.J., Barsic B., Lockhart P.B., Gewitz M.H., Levison M.E. (2015). Infective Endocarditis in Adults: Diagno-sis, Antimicrobial Therapy, and Management of Complications: A Scientific Statement for Healthcare Professionals From the American Heart Association. Circulation.

[7] Byrne J.G., Rezai K., Sanchez J.A., Bernstein R.A., Okum E., Leacche M., Balaguer J.M., Prabhakaran S., Bridges C.R., Higgins R.S.D. (2011). Surgical Management of Endocarditis: The Soci-ety of Thoracic Surgeons Clinical Practice Guideline. Ann. Thorac. Surg..

[8] Okita Y., Minakata K., Yasuno S., Uozumi R., Sato T., Ueshima K., Konishi H., Morita N., Harada M., Kobayashi J. (2016). Optimal timing of surgery for active infective en-docarditis with cerebral complications: A Japanese multicentre study. Eur. J. Cardiothorac. Surg..

[9] Angstwurm K., Borges A.C., Halle E., Schielke E., Weber J.R. (2004). Timing the valve replacement in infective endocarditis involving the brain. J. Neurol..

[10] Thuny F., Avierinos J.-F., Tribouilloy C., Giorgi R., Casalta J.-P., Milandre L., Brahim A., Nadji G., Riberi A., Collart F. (2007). Impact of cerebrovascular complications on mortality and neurologic outcome during infective endocarditis: A prospective multicentre study. Eur. Heart J..

[11] Snygg-Martin U., Gustafsson L., Rosengren L., Alsiö Å., Ackerholm P., Andersson R., Olaison L. (2008). Cerebrovascular Complications in Patients with Left-Sided Infective Endocarditis Are Common: A Prospective Study Using Magnetic Resonance Imag-ing and Neurochemical Brain Damage Markers. Clin. Infect. Dis..

[12] Wilbring M., Irmscher L., Alexiou K., Matschke K., Tugtekin S.-M. (2014). The impact of preoperative neurological events in pa-tients suffering from native infective valve endocarditis. Interact Cardiovasc. Thorac. Surg..

[13] Cahill T.J., Baddour L.M., Habib G., Hoen B., Salaun E., Pettersson G.B., Schäfers H.J., Prendergast B.D. (2017). Challenges in Infective Endocarditis. J. Am. Coll. Cardiol..

[14] Hickey G.L., Dunning J., Seifert B., Sodeck G., Carr M.J., Burger H.U., Beyersdorf F. (2015). Statistical and data reporting guidelines for theEuropean Journal of Cardio-Thoracic Surgeryand theInteractive CardioVascular and Thoracic Surgery. Eur. J. Cardio-Thoracic Surg..

[15] Ruttmann E., Willeit J., Ulmer H., Chevtchik O., Höfer D., Poewe W., Laufer G., Müller L.C. (2006). Neurological Outcome of Septic Cardioembolic Stroke After Infective Endocarditis. Stroke.

[16] Yoshioka D., Sakaguchi T., Yamauchi T., Okazaki S., Miyagawa S., Nishi H., Yoshikawa Y., Fukushima S., Saito S., Sawa Y. (2012). Impact of Early Surgical Treatment on Postoperative Neurologic Outcome for Active Infective Endocarditis Complicated by Cerebral Infarction. Ann. Thorac. Surg..

[17] Eishi K., Kawazoe K., Kuriyama Y., Kitoh Y., Kawashima Y., Omae T. (1995). Surgical management of infective endocarditis as-sociated with cerebral complications. J. Thorac. Cardiovasc. Surg..

[18] Kim Y.K., Choi C.G., Jung J., Yu S.N., Lee J.Y., Chong Y.P., Kim S.-H., Lee S.-O., Choi S.-H., Woo J.H. (2018). Effect of cerebral embolus size on the timing of cardiac sur-gery for infective endocarditis in patients with neurological complications. Eur. J. Clin. Microbiol. Infect Dis..

[19] Okonta K.E., Adamu Y.B. (2012). What size of vegetation is an indication for surgery in endocarditis?. Interact Cardiovasc. Thorac. Surg..

